# Rural family medicine as a career option among Hungarian medical students

**DOI:** 10.1080/13814788.2023.2174258

**Published:** 2023-02-16

**Authors:** András Mohos, Gergő József Szőllősi, László Róbert Kolozsvári, Jozsef Rinfel, Albert Varga, Maria Marko Kucsera, Csenge Hargittay, Peter Torzsa

**Affiliations:** a Albert Szent-Györgyi Medical School, Department of Family Medicine, University of Szeged, Szeged, Hungary; b European Rural and Isolated Practitioners Association, Paris, France; c Gottsegen Gyorgy Orszagos Kardiologiai Intezet, Budapest, 1096 Hungary; d Department of Family Medicine and Occupational Health, University of Debrecen, Debrecen, Hungary; e Institute of Primary Care, Pecs, Hungary; f Albert Szent-Gyögyi Medical School, Department of Public Health, University of Szeged, Szeged, Hungary; g Family Medicine, Semmelweiss University, Budapest, Hungary

**Keywords:** Career choice, medical student, family medicine, rural medicine, Hungary

## Abstract

**Background:**

The shortage of family physicians is a considerable challenge in Hungary. The number of vacant practices is increasing and the rural and deprived areas are more affected.

**Objectives:**

This study aimed to investigate medical students’ attitudes towards rural family medicine.

**Methods:**

The current study used a cross-sectional design with a self-administered questionnaire. Each of the four Hungarian medical universities was represented by their medical students from December 2019 to April 2020.

**Results:**

The response rate was 67.3% (*n* = 465/691). Only 5% of the participants plan to be a family doctor, 5% of the students plan to work in rural areas. On a 5-point Likert scale (1 = ‘surely not’, 5 = ‘surely yes’), half of the participants answered 1 or 2 to choose rural medical work, while 17.5% answered 4 or 5. There was a significant relationship between rural working plans and rural origin (OR = 1.97; *p* = 0.024), and the plan to work in family practice (OR = 4.90; *p* < 0.001).

**Conclusion:**

Family medicine is not a popular career option among Hungarian medical students and rural medical work is even less attractive. Medical students with a rural origin and an interest in family medicine are more likely to plan to work in rural areas. More objective information and experience need to be given to medical students about rural family medicine to increase the attractiveness of the speciality.

KEY MESSAGES

- Very few medical students think about rural family medicine as a career option.
- It is crucial to find effective strategies to stop the tendency of vacancies in practices.
- More emphasis should be put on students with rural backgrounds and rural training places to promote rural family medicine.

## Introduction

Access to the full range of essential health services is a fundamental human right but currently, there are significant inequalities and rural communities often have a disadvantage in this field [1,2]. Strong primary care is key in providing universal health coverage and contributes to an equitable, efficient and cost-effective health care system [3].

The specific characteristics of ‘Rural medicine’ vary from country to country. The differentiation between rural and urban contexts is based on various factors, such as geographic, environmental, economic, cultural, social and other characteristics [4]. The current survey did not use strict definitions, and the participants could decide what they considered to be rural, but in Hungary, for medical students, rural medicine means medical services in the countryside. In Hungary, about 30% of the population lives in rural settings. Hungary does not have geographically separated areas and an extensive territory (93,030 km^2^); economic and financial aspects play a role in regional disparities [5].

In July 2022, 677 family practices were not filled out of the 6460 (10.5%) and 516 vacant practices (76.2%) were situated in areas with fewer inhabitants than ten thousand. It means that rural areas are mostly affected by the problem of vacant practices. In parallel, this negative trend has reached the big cities; 45 vacant practices are in the capital city, Budapest [6]. The number of vacant practices has grown steadily in recent years, almost tripled in 2010–2017. There is a strong association between deprived areas and unfilled practices [7]. The number of new entrants into the GP resident training programme is insufficient to stop this trend [8].

Career choice is a multifactorial decision in medical students’ lives. The medical school, the student’s personality and preferences, the ideas about the preferred speciality and the current life situation significantly impact the decision [9]. In this article, we focus only on the impact of their attitude towards rural family medicine on career choice. Medical education plays a significant role in medical students’ lives through knowledge transfer and by shaping their attitudes. The hidden curriculum strongly influences the students’ career decisions [10]. EURACT (European Academy of Teachers in General Practice/Family Medicine) suggests that every medical faculty should have a department or unit of family medicine with a family doctor as chair and family medicine should be part of the undergraduate core curriculum [11]. There is strong evidence that the quantity of practical GP curriculum is directly correlated with medical students’ willingness to choose family medicine as a future career [12]. Rural medicine is in the same situation. EURIPA (European Rural and Isolated Practitioners Association) and EURACT state that specific rural medicine programmes must be part of medical education to increase the popularity of pastoral work [13].

The movement of family physicians from more deprived areas to developed areas causes an unequal distribution of physicians. This process also exists between countries. Since 2004, when Hungary became an EU member, healthcare professionals’ migration has become an increasing problem [7,14,15].

This study aims to describe the attitudes and motivations of Hungarian medical students towards family medicine and rural medical work and to explore the influencing factors. There were two hypotheses underlying this research: medical students of a rural origin are likelier to work in rural areas. Medical students interested in family medicine are more likely to work in rural settings.

## Methods

### Study design and participants

It was a cross-sectional survey with a self-administered paper-based questionnaire. Participation was voluntary and anonymous. Four Hungarian medical universities (Budapest, Debrecen, Szeged, and Pécs) were represented by their fourth and fifth-year medical students who attended face-to-face family medicine lectures. Because the four universities have different curricula and family medicine is included in different years, we involved the appropriate years everywhere (Table 1). Data collection was carried out from December 2019 to April 2020. After this time, it had to be stopped due to the COVID-19 pandemic. This step primarily affected the University of Pécs, where we reached a lower response rate. After receiving appropriate information about the study, 465 students decided to participate.

**Table 1.** The presence of family medicine in Hungarian universities in 2020.

Presence of family medicine	Debrecen	Pécs	Budapest	Szeged*^2^
Theory	V*.: 1 semester: seminar	IV.: 1 semester: lecture + seminar	IV.: 4 h	IV.: 2 semester – elective
Practice in family practice	IV.: 1 week	VI.: 60 h	IV.: 16 h VI.: 1 week	VI.: 1 week
Thesis or Research opportunity	Yes	Yes	Yes	Yes
Other	Elective subjects	I.: Introduction to medical communication: 1 semester Medical communication practice − 30 h of summer practice in family practice Elective subjects	I.: Introduction to patient care seminar: 1 semester VI.: 6 weeks elective practice in family practice Elective subjects	IV.: Doctor – patient communication: half-day training and half-day practice in family practice

*: year; *^2^: Compulsory family medicine lecture was introduced in 2022 in Szeged.

### Questionnaire

Data were collected with a self-developed questionnaire. There were nine questions about sociodemographic data, such as gender, age, place of origin and family role model (higher education, medical degree or family physician in the family?). Three questions were concerned with future career plans and preferred specialities. Students were asked about their planned future living place and workplace. The likelihood of rural work among participants was assessed. The first variable was ‘Do you plan to work in a rural area in the future?’ with two answer options: ‘yes’ or ‘no.’ The second variable was: ‘Do you consider it likely that you will work in a rural area in the future?’ Answers were assessed with a 5-point Likert scale (1: surely not, 2: probably not, 3: do not know, 4: probably yes, 5: surely yes). In the multivariate analysis, the categories were merged: answers 1 and 2 became ‘no,’ answers 4 and 5 became ‘yes’ and answer 3 was not used. We did not use strict definitions in the categorisation of the settlements. The capital city is Budapest but the participants could choose between the other categories. (big city, small town, or village).

### Data analyses

Data analysis was done by STATA (version 13.0, Stata Corp, College Station, TX). Descriptive statistics were performed using chi-square tests. Multivariate logistic regression models (MVA) were used to identify which factors might influence students’ future career plans focusing on the rural environment, where goodness-of-fit tests were also executed. The multivariate models consisted of two outcomes (‘X’,‘Z’). Descriptive data are presented by means/medians and standard deviations/interquartile ranges (IQR) in the case of continuous variables and as percentages in the case of categorical variables. Odds ratios describe the multivariate model results. The results were considered significant if the *p*-values derived from the statistical tests were below 0.05.

### Ethics approval

Ethics approval was received from the National Medical Research Council, Hungary, reference number 51983-2/2019/EKU.

## Results

In the relevant years, 1057 medical students studied at the four universities. In the given period, 691 participated in family medicine lectures. Of these students, 465 completed our questionnaire (67.3%). The response rate was 86.8% (*n* = 145/167) in Debrecen, 23% (*n* = 38/165) in Pécs, 63% (*n* = 131/208) in Budapest and 73.3% (*n* = 151/206) in Szeged. Sociodemographic characteristics of the sample are presented in Table 2.

**Table 2.** Sample characteristics.

Variable		Valid (*N*)	*n* (%)	
Age [mean ± SD]		465	23.5 ± 2.1 years	
Female		464	288 (62.1)	
At least one parent with a higher education degree		465	365 (79.0)	
Being a physician’s child		465	85 (18.3)	
Family or friends working in general practice		462	121 (26.2)	
Family or friends working in the preferred speciality		458	81 (17.7)	
**Come from…**		**457**		
**Urban area**	**Capital city**		**386 (84.5)**	**85 (18.6)**
	**Big city**			**160 (35.0)**
	**Small town**			**141 (30.8)**
**Rural area**			**71 (15.5)**	
University		465		
Debrecen			145 (31.2)	
Pécs			38 (8.2)	
Budapest			131 (28.2)	
Szeged			151 (32.5)	
Year		465		
Fourth			213 (45.8)	
Fifth			252 (54.2)	

The most important data are bold.

**Figure 1.**
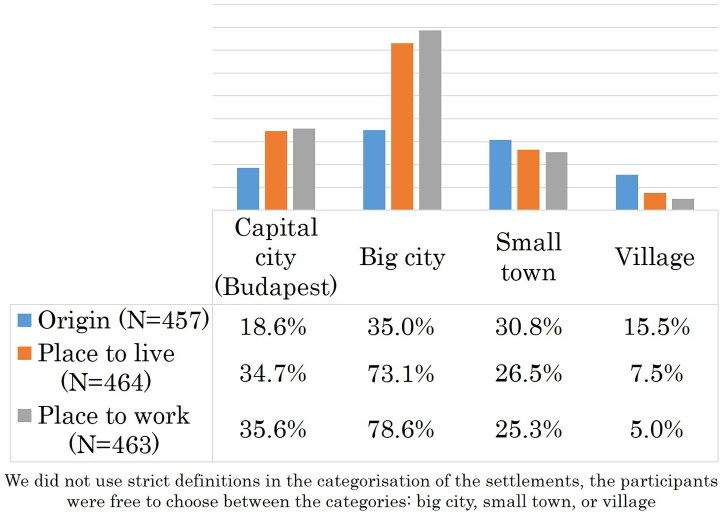
Medical students’ origin, workplace and living place plans.

Only 5% of the respondents (*n* = 23/462) plan to work as a family doctor in the future, 72% (*n* = 333/462) of them have other speciality preferences and 23% (*n* = 106/462) have not chosen their preferred speciality yet, 15.5% of the students have a rural origin. The vast majority of the participants plan to live in urban environments. The ratio of students who plan to work in rural settings is 5% (Figure 1).

**Figure 2.**
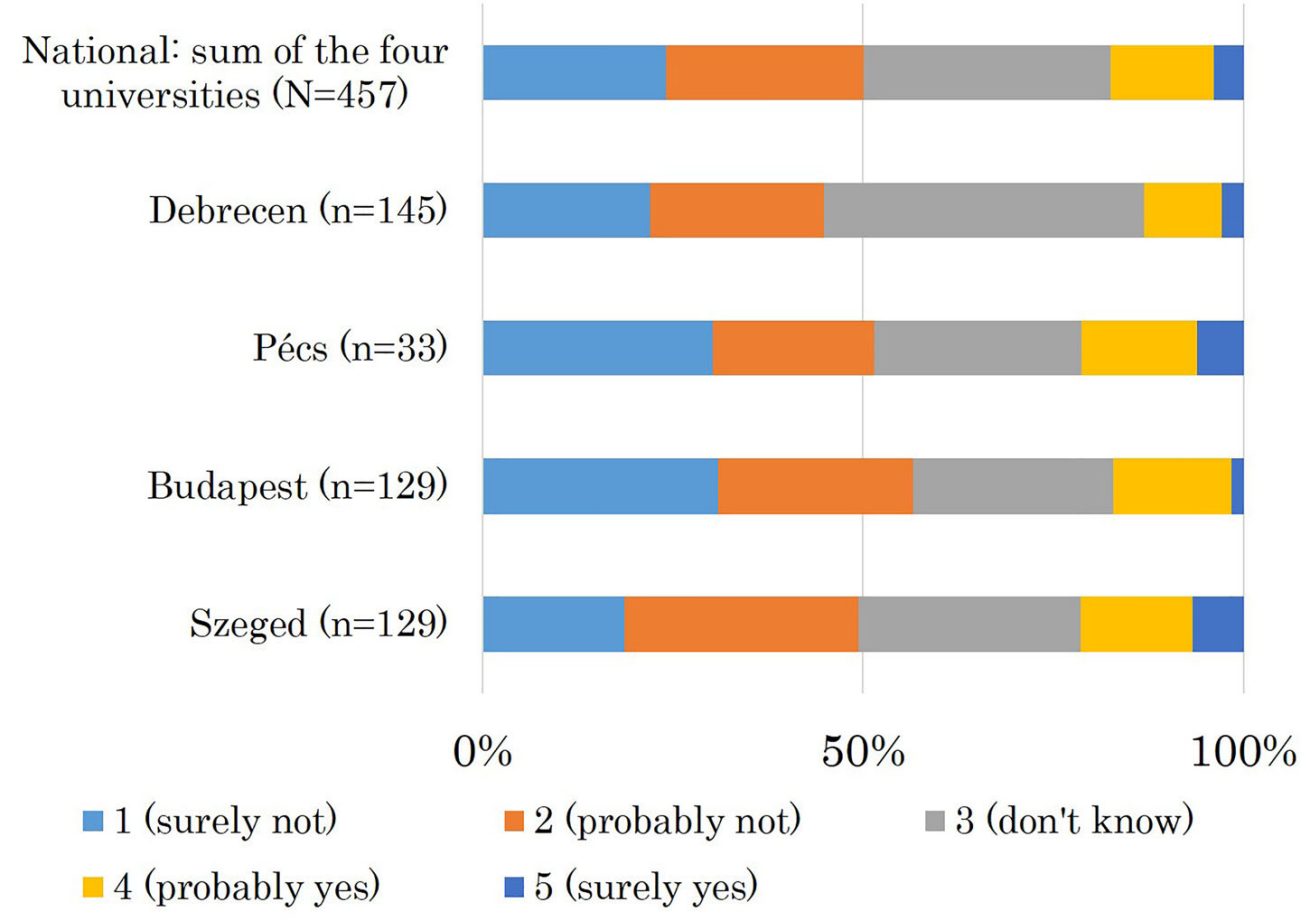
Probability of the medical students' rural work.

There was a significant positive relationship between a rural origin and rural working plans (UVA: *p* = 0.018; MVA: *OR* = 1.97; *p* = 0.024). The rural working plans had a significant correlation with family medicine as a first choice speciality (UVA: *p* < 0.001) and with the intention to work in family practice in the future (UVA: *p* < 0.001; MVA: OR = 4.90; *p* = 0.014) (Table 3); 51.5% of the students plan to work abroad after their graduation. On a 5-point Likert scale, 50.1% of the participants answered that they would ‘surely not’ or ‘probably not’ (together: ‘no’) choose rural medical work, while 17.5% answered ‘probably yes’ or ‘surely yes’ (together: ‘yes’) (Figure 2).

**Table 3.** Results of the univariate and of the multivariate analysis (UVA and MVA).

Variables	Univariate analysis							Multivariate analysis (It contains all selected variables)				
	Options	Do you plan to work in a rural area in the future?			Do you consider it likely that you will work in a rural area in the future?				Do you plan to work in a rural area in the future? (Goodness of fit: 0.2073)		Do you consider it likely that you will work in a rural area in the future? (Goodness of fit: 0.1971)	
		No	Yes	*p*	No	Yes	*p*	Options	OR	*p*	OR	*p*
University	Debrecen	110	35	0.141	126	19	0.284	Pécs/Debrecen	1.86	0.165	2.01	0.190
	Pécs	26	11		26	7		Budapest/Debrecen	0.93	0.848	1.90	0.111
	Budapest	106	25		107	22		Szeged/Debrecen	1.50	0.187	2.36	**0.018**
	Szeged	104	46		118	32						
Gender	male	125	50	0.210	142	29	0.799	female/male	0.69	0.124	0.93	0.788
	female	220	67		234	51						
Where did you grow up?	non-rural	295	90	**0.018**	323	57	**0.001**	rural/non-rural	1.97	**0.024**	3.24	**<0.001**
	rural	47	27		50	23						
Where do you plan to live in the future?	Hungary	264	85	0.278	278	67	0.094	Abroad/Hungary	1.74	**0.049**	0.66	0.249
	abroad	71	30		87	12						
At the moment, I would like to be a(*n*)…	other	337	102	**<0.001**	362	71	**0.010**	family doctor / other	1.59	0.561	0.68	0.639
	family doctor	7	14		13	8						
Long-term, I plan to work in a(*n*)…	other	334	97	**<0.001**	357	68	**0.002**	family practice / other	4.90	0.014	4.99	**0.018**
	family practice	12	20		20	12						

The results where we found significant correlation are bold.

There was a significant correlation between the probability of rural work and a rural origin (UVA: *p* = 0.001; MVA: *OR* = 3.24; *p* < 0.001), between the probability of rural work and family medicine as a first-choice speciality (UVA: *p* = 0.001) and between the probability of rural work and the plan to work in family practice in the future (UVA: *p* = 0.002; *OR* = 4.99; MVA: *p* < 0.018).

## Discussion

### Main findings

Our study found that only a few medical students think about family medicine as a career option (5%). The analysis of the reasons for low interest in family medicine is out of the scope of this work. Rural work is not attractive for students; only 5% think about a rural medical career. About one-quarter of the students would not work in rural areas in any case. However, half of the students were uncertain or showed more or less willingness towards rural work. The job is to focus on this group, give them opportunities to understand the characteristics of rural medical work better and motivate them to choose this career. The results confirmed our first hypothesis: medical students of a rural origin are more likely to plan to work in rural areas. There was a strong correlation between rural career plans and willingness to work in family practice and our second hypothesis was confirmed. Our results support that migration remains a key issue in family physicians’ recruitment. Half of the students plan to work abroad.

### Strengths and limitations

This is the first study that examined Hungarian medical students’ attitudes towards rural medical work as a career option among medical students from all four Hungarian medical universities. The sample size and response rate are acceptable and allow us to draw general conclusions about the Hungarian situation. Because of the Covid-19 Pandemic, a lower response rate was reached than initially expected. However, based on the nature of this limiting factor, we can assume that the characteristics of the participant group of students and the non-participant group of students are not different. The gender ratio supports our assumption: 62.1% (288/464) in the participant group and 63.9% (379/593) in the non-participant group. The fourth and fifth year students were at different stages of their medical studies and may have different perceptions and experience, which could be a limiting factor. The lack of a strict definition of 'rural’ can be a limiting factor. Cross-sectional data cannot be used to infer causality. Only a few of the medical students are interested in family medicine and rural medical work as a future career option; therefore we could not describe the unique characteristics of this group.

### Comparison with existing literature

In our previous studies, 0–3.9% of medical students planned to be a family doctor definitely and 12.1–19.2% thought about it [16]. Worldwide, healthcare systems face similar challenges and the popularity of primary care work is not high enough among medical students. Naimer et al. found that 19% of Israeli students are interested in family medicine [17]. In Germany, 12.3% of the students plan to opt for family medicine [18]. Girasek et al. found that in 2008, only 0.8% of Hungarian resident doctors planned to work in places with fewer inhabitants than 10,000 [19]. In our previous research in 2016, students with primary care orientation were 28 times more likely to work in rural areas than the students who would have liked to work in secondary care [16], 21% of Argentinians, 44% of the Indians and 42% of the Australian medical students want to work in rural areas. In these countries, rural medicine gets more emphasis during medical education, students get more impulses and experience from this field and undergraduate rural is significantly associated with long-term rural work [20–23]. Studies show that financial and economic incentives and rural practice experience during the medical years could drive these students to rural pathways. However, not only financial aspects but also lifestyle factors play an essential role in the decision [24,25]. The Hungarian government supports rural employment with different tender opportunities but it does not seem to be motivating enough for doctors or medical students. A meta‑analysis from 2020 states that rural exposure during medical education increases the likelihood of future rural practice by more than four times on average [26]. In the case of complex educational programmes, the background of medical students, their interactions with rural communities and many other factors also play an important role. The rural community-based medical education programme in Thailand and the many Australian rural education programmes are successful examples of initiatives to solve the rural human resource crisis [27,28]. Győrffy et al. found that in 2016 almost 40% of the participating fifth and sixth-year medical students planned to work abroad as a doctor. Based on these, we can state that the tendency did not change significantly. [14].

### Implications for practice and policy

Undergraduate education has a crucial role in specialisation. Different career choice-supporting resources and programmes could positively impact students’ engagement with career planning [29]. It seems worthwhile to handle the challenges of the rural human resource crisis and human resource recruitment in family medicine together. Training more general practitioners could be important in addressing the rural doctor shortage. We recommend increasing the presence of family medicine and rural medical work in the curriculum. Creating strategies to involve more rural students in medical universities should be considered. Furthermore, it is vital to recognise what factors determine urban students, who are in the majority, to choose rural medical work. Moreover, the universities, medical organisations, government, and the media are also responsible for authentic informing of medical students about their career choice. The shortage of rural family physicians is not an exclusively Hungarian issue. Awareness of different countries’ challenges or solution proposals could trigger better local initiatives in every country or international cooperation.

## Conclusion

Human resource recruitment in rural primary care is an unsolved problem. It is crucial to find enough highly qualified medical professionals with primary care orientation and it needs young doctors who are interested in rural medical work. Only a few medical students consider rural family medicine as a career option in Hungary. Implementing international programmes and adapting them to local conditions would be beneficial. To promote rural medical work and family medicine as a career, more emphasis should be put on medical students with rural backgrounds and rural training places.

## Data Availability

The datasets used and/or analysed during the current study are available from the corresponding author upon reasonable request.
